# Comparative Evaluation of Visual Performance and Patient Satisfaction following Cataract Surgery: A Retrospective Analysis of an Extended Depth-of-Focus Intraocular Lens and a Diffractive Multifocal Lens with Extended Depth of Focus

**DOI:** 10.3390/jcm12237368

**Published:** 2023-11-28

**Authors:** Kwang Eon Han, Ji Eun Lee

**Affiliations:** 1 Department of Ophthalmology, Pusan National University Yangsan Hospital, Pusan National University School of Medicine, Yangsan 50612, Republic of Korea; kehanoph@pusan.ac.kr; 2 Research Institute for Convergence of Biomedical Science and Technology, Pusan National University Yangsan Hospital, Yangsan 50612, Republic of Korea

**Keywords:** cataract surgery, visual acuity, intraocular lens, Synergy^®^, Symfony^®^

## Abstract

(1) Background: Cataract surgery has evolved significantly with the development of multifocal and extended depth-of-focus intraocular lenses (IOLs), driven by increasing patient desire for spectacle independence. (2) Methods: This retrospective, single-center study conducted on 86 eyes from 59 patients aged 40–80 years compared the performance and patient satisfaction of Symfony^®^ and Synergy^®^ (Johnson & Johnson Vision) IOLs during a follow-up of 1 year postsurgery. Uncorrected and corrected distance, intermediate, and near visual acuities were assessed (UDVA, CDVA, UIVA, CIVA, UNVA, and CNVA, respectively). (3) Results: Although both IOLs demonstrated a commendable distance VA, Synergy^®^ outperformed in near VA (UNVA, *p* = 0.040; CNVA, *p* = 0.014), and Symfony^®^ slightly excelled in intermediate VA (UIVA, *p* = 0.014; CIVA, *p* = 0.040). The defocus curve of Synergy^®^ maintained a higher VA even at −4 D with a smoother curve and a broad landing zone. Although the optical quality assessments were similar, Symfony^®^ had a nonsignificant edge. Patients indicated higher satisfaction and reduced reliance on spectacles with Synergy^®^ despite more frequent reports of glare and halos. (4) Conclusions: These findings highlight the importance of personalized IOL selection in cataract surgery, which must be customized to apply the distinctive advantages of each IOL to address the unique visual requirements and lifestyle of patients.

## 1. Introduction

Cataract is a significant cause of global blindness, particularly in developing countries [1]. Despite the widespread availability of cataract surgery, the burden of cataract blindness and visual impairment remains significant, particularly in low- and middle-income countries. Globally, in 2020, an estimated 79 million individuals over the age of 50 suffered from moderate to severe visual impairment (presenting visual acuity <6/18 to 3/60) due to cataracts. These estimates represent a 30% increase in cataract blindness and a 93% increase in moderate to severe visual impairment compared to the year 2000 [2]. Traditionally, cataract surgery entails replacing the natural lens with an artificial lens, primarily focused on either distance or near vision. However, the evolving preference of patients is to achieve spectacle independence for intermediate and near distances, even though there may be a diversity in patient expectations and potential socioeconomic factors influencing their preferences [3]. Novel multifocal intraocular lenses (IOLs) have emerged, allowing patients who have undergone cataract surgery to experience more spectacle independence [4]. However, this pursuit of independence can result in variations in optical quality because of photic phenomena, higher-order aberrations, reduced contrast sensitivity (CS), and diminished visual acuity (VA) between individual focal points as they distribute available light [5]. These optical phenomena are likely to affect patients’ postoperative satisfaction and daily functioning.

To address these challenges, a variety of EDOF and multifocal IOLs have been introduced. In this study, we compared two IOLs: one EDOF IOL and another with added multifocal elements. Symfony^®^ (Johnson & Johnson Vision, Santa Ana, CA, USA), an extended depth-of-focus (EDOF) IOL, was introduced in 2014, offering excellent intermediate vision, although near vision remains relatively suboptimal [6]. Synergy^®^ (Johnson & Johnson Vision) has patented diffractive surfaces and combines multifocal and EDOF technologies to create a single elongated focal point, which grants patients a wider range of clear vision without necessitating reading glasses [7,8]

Currently, only one study has compared Symfony^®^ and Synergy^®^ IOLs and reported 3-month postoperative visual outcomes and optical qualities in only 38 eyes [9]. The purpose of this study was to add long-term results with more patients to the current literature by evaluating visual outcomes at various distances (distance, intermediate, and near); defocus curves; and optical qualities, including CS, high-order aberrations, photic phenomena, and spectacle dependency, over a 1-year postsurgery period.

## 2. Materials and Methods

### 2.1. Study Participants

This was a retrospective, single-center, comparative study of two multifocal IOLs. The study adhered to the principles of the Declaration of Helsinki and obtained approval from the Institutional Review Board of Pusan National University Yangsan Hospital (IRB no. 55-2023-036). To ensure patient anonymity, any personally identifiable information was removed from the data before analysis. This retrospective cohort study encompassed 86 eyes from 59 patients with cataract who had undergone cataract surgery with Symfony^®^ or Synergy^®^ insertion between January 2020 and December 2021 at the Department of Ophthalmology, Pusan National University Yangsan Hospital, and were followed up for at least 12 months postsurgery. The study included patients aged 40–80 years with regular corneal astigmatism and no other major ocular pathologies. Exclusion criteria encompassed relevant ophthalmic conditions that could alter or affect outcomes, history of ocular trauma or surgery, systemic or ocular medications, or irregular corneal astigmatism potentially impeding postoperative visual results.

Preoperatively, all patients underwent ophthalmic examinations, including corrected distance VA (CDVA), slit-lamp examination, intraocular pressure (IOP) measurement using Goldmann applanation tonometry, and fundoscopy. Axial length, anterior chamber depth, average keratometry, and IOL power were obtained using IOL Master (Carl Zeiss Meditec, Dublin, CA, USA). Corneal thickness and average keratometry were calculated using iTrace incorporating corneal topography (Tracey Technologies, Houston, TX, USA).

At the 1-year postoperative visit, uncorrected distance (at 4 m) VA (UDVA), CDVA, uncorrected intermediate (at 66 cm) VA (UIVA), corrected intermediate VA (CIVA), uncorrected near (at 33 cm) VA (UNVA), and corrected near VA (CNVA) were measured. All VA results were converted to the logarithm of the minimum angle of resolution (logMAR) for statistical analysis.

Monocular defocus curves were obtained 1 year after surgery using defocusing lenses with a power range of 1.0 to −4.0 D in 0.5 D steps. Lenses were inserted into a test frame to correct refractive errors.

### 2.2. Surgical Technique

All operations were performed by a single expert surgeon (J.E.L.), who made a 2.8 mm temporal incision in the limbus of the cornea. Viscoelastic material (DisCoVisc; Alcon, Fort Worth, TX, USA) was injected into the anterior chamber. A 5 mm capsulorhexis, slightly smaller than the optical portion of the intraocular lens, was created. After performing hydrodissection or hydrodelineation with irrigation fluid, the nucleus was phacoemulsified and the cortex was aspirated with an ultrasound phacoemulsification device (Infinity, Alcon). Subsequently, the multifocal IOL was inserted into the capsular bag using an injector system. Viscoelastic material was then removed using an irrigation/aspiration device, and a clear corneal incision was made with stromal hydration.

### 2.3. Optical Quality Assessment

Three months postoperatively, CS was measured at 1.5, 3, 6, 12, and 18 cycles per degree (cpd) under mesopic (~3 cd/m^2^) conditions using a CSV-1000 chart (Vector Vision, Greenville, OH, USA). Results were converted into log units for statistical analysis using a specific table for CSV-1000. The modulation transfer function (MTF), root mean square (RMS), and Strehl ratio (SR) were measured 1 year postoperatively using an iTrace aberrometer (Tracey Technologies Corp., Houston, TX, USA) under 3.0 mm pupil conditions. MTF curves were calculated at 0, 5, 10, 15, 20, 25, and 30 cpd. RMS values were measured for total-order aberrations, coma, spherical aberrations, and trefoil.

### 2.4. Patient Satisfaction Questionnaire

A questionnaire was administered 1 year after surgery to assess overall patient satisfaction, the presence of glare or halos, and dependence on glasses. Overall patient satisfaction was rated on a 5-point scale ranging from “very dissatisfied” (1 point) to “very satisfied” (5 points) (Table A1).

### 2.5. Statistical Analysis

All statistical analyses were performed using SPSS Statistics for Windows (version 27.0; IBM Corp., Armonk, NY, USA). The Kolmogorov–Smirnov test was used to check the normality of the data distribution. The two groups were compared using Pearson’s chi-square test and Student’s *t*-test. A *p*-value of <0.05 was considered statistically significant (*p* < 0.05). Data are presented as mean ± standard deviation.

## 3. Results

A total of 44 eyes (31 patients; mean age, 53.6 ± 14.4 years; age range, 21–73 years) were implanted with Symfony^®^, whereas 42 eyes (28 patients, mean age, 56.4 ± 4.7; age range, 46–62 years) received Synergy^®^ implants. In the Symfony^®^ and Synergy^®^ groups, 17 (54.8%) and 12 (42.9%) patients were female, respectively. No significant differences were observed in preoperative characteristics, including corrected VA, axial length, corneal thickness, anterior chamber depth, average keratometry, IOL power, and IOP, between the two groups (Table 1). One expert surgeon (J.E.L.) performed all surgeries, and no intraoperative or postoperative complications were recorded for either surgical procedure.

The postoperative visual acuities for distance, intermediate, and near vision at 1 year are presented in Table 2. No significant difference was noted in the UDVA or CDVA between the two groups (*p* = 0.583 and *p* = 0.452, respectively). IVAs showed more improvement for Symfony^®^ than for Synergy^®^ in both CIVA (*p* = 0.107) and UIVA (*p* = 0.589) but without statistical significance. However, Synergy^®^ outperformed Symfony^®^ in NVA, both in CNVA (*p* = 0.040) and UNVA (*p* = 0.014).

**Figure 1:**
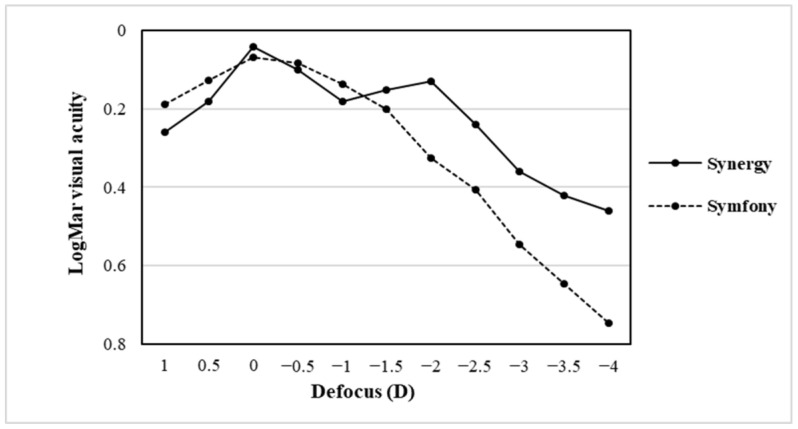
Mean monocular defocus curves obtained from Synergy^®^ and Symfony^®^ groups. *LogMAR* logarithm of the minimal angle of resolution; *D*, diopters.

The defocus curves determined 1 year after surgery are shown in Figure 1. Both IOLs showed the best VA at 0 D. Symfony^®^ maintained a logMAR of ≥0.3 from + 1.0 to −1.5 D, whereas Synergy^®^, maintained this level of VA from + 1.0 to −2.5 D. Symfony^®^ maintained VA of a logMAR of ≥0.12 from 0 to −0.5 D, whereas Synergy^®^ maintained it from 0 to −2.0 D. Notably, although Synergy^®^ showed some dips in VA in the intermediate range (−0.5 to −1.5 D), the defocus curve of Synergy^®^ consistently maintained VA superior to logMAR 0.4, even at −3.0 D, and it showed a smoother curve with a wider landing area than Symfony^®^.

**Figure 2:**
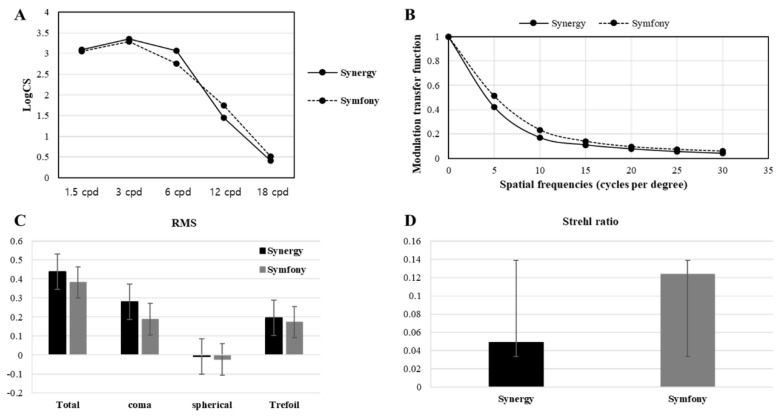
Optical quality parameters obtained from Synergy^®^ and Symfony^®^ groups. (**A**) Contrast sensitivity (CS); (**B**) modulation transfer function (MTF); (**C**) root mean score (RMS); (**D**) Strehl ratio. The vertical bars represent the standard deviation at each frequency.

At the last follow-up, optical qualities (CS, MTF, RMS, and SR) were assessed. CS curves with distance correction are shown in Figure 2A. In Symfony^®^, the CS was 3.02 ± 0.68 (1.5 cpd), 3.28 ± 0.69 (3 cpd), 2.81 ± 1.49 (6 cpd), 1.96 ± 2.05 (12 cpd), and 0.93 ± 2.76 (18 cpd). For Synergy^®^, these values were 3.05 ± 0.18 (1.5 cpd), 3.36 ± 0.22 (3 cpd), 3.14 ± 0.53 (6 cpd), 1.66 ± 1.34 (12 cpd), and 0.35 ± 0.85 (18 cpd). Although Synergy^®^ outperformed Symfony^®^ in terms of CS curve at 6 cpd, the CS curve was higher with Symfony^®^ at 12 and 18 cpd without significant differences (*p* ≥ 0.25). MTF curves at different spatial frequencies with a 3.0-mm pupil are shown in Figure 2B. The mean MTF value for each IOL was 0.24 ± 0.09 (Symfony^®^) and 0.24 ± 0.06 (Synergy^®^). Although no significant difference (*p* ≥ 0.17) was observed between them for all spatial frequencies, Symfony^®^ demonstrated superior performance compared to Synergy^®^. RMS values of high-order aberrations (total, coma, spherical, and trefoil) are presented in Figure 2C. Synergy^®^ displayed a trend of increased aberrations compared to Symfony^®^, but the difference was not significant (*p* ≥ 0.28). The SR of the two IOLs are shown in Figure 2D. The mean SR for each IOL was 0.12 ± 0.12 (Symfony^®^) and 0.05 ± 0.03 (Synergy^®^) without significant differences (*p* ≥ 0.33). Therefore, Symfony^®^ outperformed Synergy^®^ in all optical quality parameters, although the differences were not statistically significant.

**Figure 3:**
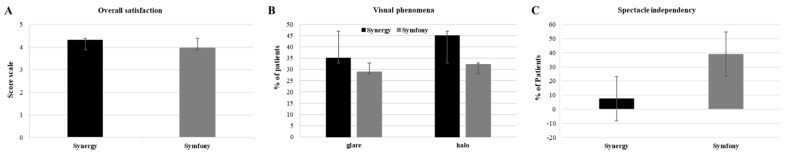
Postoperative questionnaire obtained from the Synergy^®^ and Symfony^®^ groups. (**A**) Overall satisfaction questionnaire; (**B**) visual phenomena questionnaire; (**C**) spectacle independency questionnaire. The vertical bars represent the standard deviation at each frequency.

Postoperative questionnaires were administered during the final follow-up visit. Both groups reported similar overall satisfaction levels (Figure 3A). Synergy^®^ was associated with a significantly higher frequency of visual symptoms (glare and halo) (*p* = 0.046 and 0.021, respectively) (Figure 3B). A questionnaire that assessed postoperative spectacle dependency revealed a significantly higher spectacle dependence in Symfony^®^ compared to Synergy^®^ (*p* = 0.041) (Figure 3C).

## 4. Discussion

EDOF IOLs have been extensively studied and have demonstrated positive clinical outcomes. These lenses are associated with improved visual and patient-reported outcomes in cataract surgery, offering continuous focus across distances and enhancing near and intermediate vision [10]. Additionally, EDOF IOLs aim to reduce photic phenomena, such as glare and halos, which can occur with other types of lenses [11]. Studies have also compared EDOF IOLs to multifocal IOLs, demonstrating their effectiveness in providing clear distance vision and improved intermediate vision [12,13]. Based on these EDOF technologies, Symfony^®^ was launched in 2016. Several studies have reported that Symfony^®^ lenses provide enhanced intermediate vision, superior depth of focus (DOF), greater spectacle independence, and overall satisfaction [13,14,15]. Recently, with the release of Synergy^®^, studies have investigated the associated vision, optical quality, patient satisfaction, and spectacle independence [16,17]. Only one study has compared Symfony^®^ and Synergy^®^ using similar criteria [9]. We aimed to compare VA, DOF, optical qualities, and patient experience evaluations over a longer postoperative follow-up period with a larger number of patients.

Both IOLs demonstrated excellent distance-VA outcomes with no significant differences between them. However, a significant difference was present between intermediate and near VA. This study showed that Symfony^®^ outperformed Synergy^®^ for UIVA and CIVA, whereas Synergy^®^ was better for UNVA and CNVA. This suggests that the choice between the two IOLs for implantation may depend on the specific visual needs of each patient. Earlier studies investigating the distance VA of Symfony^®^ and Synergy^®^ have reported satisfactory results for vision [9,18,19,20]. Our results are apparently consistent with their findings in both CDVA and UDVA. Several studies comparing Symfony^®^ and Synergy^®^ with other IOLs have reported UIVA values in the range of 0.22 ± 0.12 to 0.08 ± 0.14 for Symfony^®^ [9,19,21] and 0.05 ± 0.09 to 0.01 ± 0.04 for Synergy^®^ [9,20,22]. CIVA values were reported to be in the range of 0.12 ± 0.05 to 0.04 ± 0.08 for Symfony^®^ [23,24,25] and 0.02 ± 0.04 to 0.01 ± 0.107 for Synergy^®^ [8,18]. However, Moshirfar et al. [26], who compared three multifocal or EDOF lenses, reported that Symfony^®^ had a higher UIVA than Synergy^®^ (0.09 and 0.22, respectively). The results of our study also showed that both Symfony^®^ and Synergy^®^ had better visual outcomes compared to those in previous studies for intermediate vision, with Symfony^®^ showing slightly better visual outcomes than Synergy^®^ without statistical significance [26]. Previous studies have reported UNVA values in the range of 0.27 ± 0.11 to 0.41 ± 0.09 [9,27,28] for Symfony^®^ and 0.04 ± 0.09 to 0.10 ± 0.07 for Synergy^®^ [17,22]. CNVA values were reported in the range of 0.33 ± 0.11 to 0.16 ± 0.15 for Symfony^®^ [18,23,25] and 0.03 ± 0.11 to 0.00 ± 0.03 for Synergy^®^ [8,18]. Consistent with other studies, Synergy^®^ surpassed Symfony^®^ in both uncorrected and corrected near vision. Furthermore, we could achieve better near-visual outcomes than those in other studies during the 1-year follow-up period.

The defocus curve test is a valuable tool for assessing the continuous visual function at different distances after implantation of IOLs, especially functional ones [29]. We adopted the blur criterion at 0.3 and 0.12 logMAR. Synergy^®^ had a wider DOF range in our study than Symfony^®^. Synergy^®^ maintained a VA of 0.3 logMAR or better from +1.0 to −2.5 D and a VA of 0.12 logMAR or better from 0 to −2.0 D. Assessing the subjective depth of focus of Synergy^®^, Palomino-Bautista et al. [18] reported that the VA of Synergy^®^ was maintained at a level of 0.12 logMAR or better over a range of +0.5 to −3.0 D and at a level of 0.3 logMAR or better over a range of +1.0 to −4.0 D. Shin et al. [9] revealed Synergy^®^ had a sustained VA of 0.3 logMAR or better from +1.0 to −4.0 D and a VA of 0.12 logMAR or better from 0.5 to −2.5 D. Although these results were superior to those of our study, which had a slightly narrower DOF, Synergy^®^ in this study also had a smoother defocus curve with high VA even at −4.0 D than that for Symfony^®^.

Optical quality is a subjective measure of how well an optical system performs and can only be indirectly inferred from objective metrics, such as wavefront error measurements and visual quality metrics, or functional data, such as VA and CS. Two studies comparing the CS of Symfony^®^ and other multifocal IOLs reported that Symfony^®^ showed better mesopic CS [30,31]. Unfortunately, published data directly comparing the CS of Symfony^®^ and Synergy^®^ IOLs are not available. In this study, we compared both and showed that Symfony^®^ and Synergy^®^ had similar values, with a higher value at each point in Symfony^®^ without any significance. Khoramnia et al. [32] reported exact mesopic CS values for Symfony^®^, which were 1.56 ± 0.20, 1.44 ± 0.25, 0.92 ± 0.38, and 0.44 ± 0.37 at illumination levels of 3.0, 6.0, 12.0, and 18.0, respectively, and were similar to our results. iTrace measures the objective optical quality of an IOL using a retinal image obtained from a ray-tracing aberrometer system [33]. This equipment enabled us to obtain more accurate ocular aberration measurements for multifocal IOLs because the reflected image is less affected by light scattering [34]. MTF, RMS, and SR values, which are representative indicators of optical quality, closely correlated with wavefront aberration, reflecting the extent of nonuniformity in the optical wavefront. Symfony^®^ demonstrated superior performance compared to Synergy^®^, consistent with prior studies, indicating that the optical quality of Synergy^®^ may be compromised by light splitting and distortion from the combination of EDOF and bifocal technologies [9,28,35].

In this study, the incidence of glare and halo were significantly higher with Synergy^®^. One possible explanation for this is that a large number of diffractive steps may be responsible for glare and halo; Symfony^®^ has 9 diffractive steps, whereas Synergy^®^ has 15. This difference in the diffractive step number can explain the results in the photic phenomena as described above. However, despite the higher incidence of photic phenomena (halo and glare), patients who received Synergy^®^ reported significantly better satisfaction and greater spectacle independence than those who received Symfony^®^. This may be because Synergy^®^ showed a greater increase in both distance- and near-VA outcomes than Symfony^®^, providing a more pronounced perceived effect in addressing presbyopia for patients.

This study has several limitations, primarily the retrospective design and absence of randomization, as patients chose the IOL based on their personal preferences and economic circumstances. Furthermore, the findings of our single-center study may not be generalizable to a larger population because of the specific demographic and clinical characteristics of the participants from a single location. Additionally, the age range in our study was wide and there might be age-related variability in outcomes. Finally, the definitive determination of photic phenomena is challenging as many IOL studies employ nonvalidated questionnaires for assessing patient-related outcomes, and questionnaire variations can exist.

In conclusion, Symfony^®^ showed improved intermediate vision, optical quality, and definitive determination of photic phenomena, whereas Synergy^®^ demonstrated better near vision, a smoother defocus curve, and increased spectacle independence. The selection of an IOL for cataract surgery should be tailored to individual visual requirements and lifestyle of the patient, taking into account the potential advantages offered by each IOL type to enhance their quality of life.

## Figures and Tables

**Table 1:** Preoperative patient characteristics of two groups.

Parameter	Symfony^®^	Synergy^®^	*p*-Value
Eyes, N	44	42	N/A
Female, N	25(56.8%)	19(45.2%)	0.098 *
Age (years)	53.6 ± 14.4 (21 to 73)	56.4 ± 4.7 (46 to 62)	0.324 ^†^
CDVA (logMAR)	0.50 ± 0.32 (0 to 1.6)	0.53 ± 0.21 (0.2 to 0.6)	0.804 ^†^
Axial length (mm)	23.7 ± 1.19 (22.4 to 27.2)	24.1 ± 1.58 (22.7 to 26.9)	0.360 ^†^
Corneal thickness (µm)	550 ± 32.4 (486 to 666)	530 ± 53.9 (444 to 583)	0.318 ^†^
Anterior chamber depth (mm)	3.27 ± 0.43 (2.47 to 4.30)	3.39 ± 0.34 (3.00 to 3.99)	0.479 ^†^
Avg K (D)	44.0 ± 1.49 (39.3 to 45.8)	44.4 ± 1.34 (41.4 to 45.4)	0.461 ^†^
IOL power (D)	20.8 ± 3.23 (9.5 to 24.5)	17.9 ± 5.86 (10.5 to 24.5)	0.210 ^†^
Intraocular pressure (mmHg)	15.8 ± 2.81 (10 to 22)	16.4 ± 3.16 (12 to 21)	0.586 ^†^

N/A = not applicable; CDVA = corrected distance visual acuity; LogMAR = logarithm of the minimum angle of resolution; Avg K = average keratometry; IOL = intraocular lens; D = diopter; * chi-square test; ^†^ Student’s *t*-test.

**Table 2:** Postoperative visual outcomes of the two groups.

Parameter	Symfony^®^	Synergy^®^	*p*-Value
UDVA (LogMAR)	0.15 ± 0.22 (−0.2 to 0.7)	0.10 ± 0.27 (−0.2 to 0.5)	0.583
CDVA (LogMAR)	0.04 ± 0.21 (−0.2 to 0.5)	0.10 ± 0.27 (−0.2 to 0.5)	0.452
UIVA (LogMAR)	−0.15 ± 0.19 (−0.3 to 0.5)	0.18 ± 0.09 (0.1 to 0.3)	<0.001 *
CIVA (LogMAR)	−0.13 ± 0.24 (−0.3 to 0.8)	0.04 ± 0.05 (0 to 0.1)	0.003 *
UNVA (LogMAR)	0.23 ± 0.20 (−0.3 to 0.5)	0.05 ± 0.08 (−0.1 to 0.1)	0.014 *
CNVA (LogMAR)	0.16 ± 0.19 (−0.3 to 0.5)	0.01 ± 0.08 (−0.1 to 0.1)	0.040 *

UDVA, uncorrected distance visual acuity; CDVA, corrected distance visual acuity; UIVA, uncorrected intermediate visual acuity; CIVA, corrected intermediate visual acuity; UNVA, uncorrected near visual acuity; CNVA, corrected near visual acuity; LogMAR, logarithm of the minimum angle of resolution; * *p* < 0.05.

## Data Availability

All data pertaining to the study are described in the manuscript.

## Appendix A

**Table A1:** Questionnaire administered after multifocal intraocular lens implantation.

1. Overall satisfaction with the surgery:				
Very satisfied (5)	Satisfied (4)	Neutral (3)	Dissatisfied (2)	Very dissatisfied (1)
2. Do you need glasses after the surgery?				
Yes ( )			No ( )	
3. If you need to wear glasses, when do you usually need them?				
Distance ( )			Near ( )	
4. Do you have any discomfort?				
Yes ( )			No ( )	
5. If you have any discomfort, please list them. (Multiple answers are possible.)				
Glare ( )	Halos ( )	Starbursts ( )	Difficulty seeing text ( )	Other ( )
6. Would you recommend this surgery to others?				
Yes ( )			No ( )	

## References

[1] Lee C.M., Afshari N.A. (2017). The global state of cataract blindness. Curr. Opin. Ophthalmol..

[2] Cicinelli M.V., Buchan J.C., Nicholson M., Varadaraj V., Khanna R.C. (2023). Cataracts. Lancet.

[3] Greenstein S., Pineda R. (2017). The quest for spectacle Independence: A comparison of multifocal intraocular lens implants and pseudophakic monovision for patients with presbyopia. Semin. Ophthalmol..

[4] De Medeiros A.L., de Araújo Rolim A.G., Motta A.F.P., Ventura B., Vilar C., Chaves M., Carricondo P., Hida W. (2017). Comparison of visual outcomes after bilateral implantation of a diffractive trifocal intraocular lens and blended implantation of an extended depth of focus intraocular lens with a diffractive bifocal intraocular lens. Clin. Ophthalmol..

[5] Singh B., Sharma S., Dadia S., Bharti N., Bharti S. (2020). Comparative evaluation of visual outcomes after bilateral implantation of a diffractive trifocal intraocular lens and an extended depth of focus intraocular lens. Eye Contact Lens..

[6] Law E.M., Aggarwal R.K., Buckhurst H., Kasaby H.E., Marsden J., Shum G., Buckhust P.J. (2021). One-year postoperative comparison of visual function and patient satisfaction with trifocal and extended depth of focus intraocular lenses. Eur. J. Ophthalmol..

[7] Ozturkmen C., Kesim C., Gunel Karadeniz P., Sahin A. (2021). Comparative analysis of a new hybrid EDOF-multifocal diffractive intraocular Lens with a trifocal diffractive intraocular Lens. Eur. J. Ophthalmol..

[8] Dick H.B., Ang R., Corbett D., Hoffmann P., Tetz M., Villarrubia A., Palomino C., Castillo-Gomez A., Tsai L., Thomas E.K. (2022). Comparison of 3-month visual outcomes of a new multifocal intraocular lens versus a trifocal intraocular lens. J. Cataract. Refract. Surg..

[9] Shin D.E., Lee H., Kim T.I., Koh K. (2022). Comparison of visual results and optical quality of two presbyopia-correcting intraocular lenses: TECNIS symfony versus TECNIS synergy. Eur. J. Ophthalmol..

[10] Gawęcki M., Prądzyńska N., Kiciński K., Ratajczak A., Karska-Basta I., Grzybowski A. (2023). Patient reported outcomes after implementation of an enhanced depth of focus intraocular lens with low postoperative myopia. Adv. Ophthalmol. Pract. Res..

[11] Megiddo-Barnir E., Alió J.L. (2023). Latest Development in Extended Depth-of-Focus Intraocular Lenses: An Update. Asia Pac. J. Ophthalmol..

[12] Kondylis G., Klavdianou O., Palioura S. (2019). Multifocal and extended depth of focus intraocular lenses. Ann. Eye Sci..

[13] Guo Y., Wang Y., Hao R., Jiang X., Liu Z., Li X. (2021). Comparison of Patient Outcomes following Implantation of Trifocal and Extended Depth of Focus Intraocular Lenses: A Systematic Review and Meta-Analysis. J. Ophthalmol..

[14] Tan J., Qin Y., Wang C., Yuan S., Ye J. (2019). Visual quality and performance following bilateral implantation of TECNIS Symfony intraocular lenses with or without micro-monovision. Clin. Ophthalmol..

[15] Sachdev G.S., Ramamurthy S., Sharma U., Dandapani R. (2018). Visual outcomes of patients bilaterally implanted with the extended range of vision intraocular lens: A prospective study. Indian. J. Ophthalmol..

[16] De Rojas J.O., Sandoval H.P., Potvin R., Solomon K.D. (2023). Visual Outcomes, Quality of Vision, Patient Satisfaction and Spectacle Independence after Bilateral Implantation of the Synergy™ Intraocular Lens. Clin. Ophthalmol..

[17] Moshirfar M., Stapley S.R., Corbin W.M., Bundogji N., Conley M., Darquea I.M., Ronquillo Y.C., Hoopes P.C. (2022). Comparative Visual Outcome Analysis of a Diffractive Multifocal Intraocular Lens and a New Diffractive Multifocal Lens with Extended Depth of Focus. J. Clin. Med..

[18] Palomino-Bautista C., Sánchez-Jean R., Carmona-Gonzalez D., Piñero D.P., Molina-Martín A. (2021). Depth of field measures in pseudophakic eyes implanted with different type of presbyopia-correcting IOLS. Sci. Rep..

[19] Jeon Y.J., Yoon Y., Kim T.I., Koh K. (2021). Comparison between an intraocular lens with extended depth of focus (Tecnis symfony ZXR00) and a new monofocal intraocular lens with enhanced intermediate vision (Tecnis eyhance ICB00). Asia Pac. J. Ophthalmol..

[20] Ribeiro F.J., Ferreira T.B., Silva D., Matos A.C., Gaspar S. (2021). Visual outcomes and patient satisfaction after implantation of a presbyopia-correcting intraocular lens that combines extended depth-of-focus and multifocal profiles. J. Cataract. Refract. Surg..

[21] Kohnen T., Böhm M., Hemkeppler E., Schönbrunn S., DeLorenzo N., Petermann K., Herzog M. (2019). Visual performance of an extended depth of focus intraocular lens for treatment selection. Eye.

[22] Ferreira T.B., Ribeiro F.J., Silva D., Matos A.C., Gaspar S., Almeida S. (2022). Comparison of refractive and visual outcomes of 3 presbyopia-correcting intraocular lenses. J. Cataract. Refract. Surg..

[23] Ganesh S., Brar S., Pawar A., Relekar K.J. (2018). Visual and Refractive Outcomes following Bilateral Implantation of Extended Range of Vision Intraocular Lens with Micromonovision. J. Ophthalmol..

[24] Miháltz K., Szegedi S., Steininger J., Vécsei-Marlovits P.V. (2022). The relationship between patient satisfaction and visual and optical outcome after bilateral implantation of an extended depth of focus multifocal intraocular lens. Adv. Ophthalmol. Pract. Res..

[25] Ang R.E., Picache G.C.S., Rivera M.C.R., Lopez L.R.L., Cruz E.M. (2020). A Comparative Evaluation of Visual, Refractive, and Patient-Reported Outcomes of Three Extended Depth of Focus (EDOF) Intraocular Lenses. Clin. Ophthalmol..

[26] Moshirfar M., Stoakes I.M., Theis J.S., Porter K.B., Santos J.M., Martheswaran T., Payne C.J., Hoopes P.C. (2023). Assessing Visual Outcomes: A Comparative Study of US-FDA Premarket Approval Data for Multifocal and EDOF Lens Implants in Cataract Surgery. J. Clin. Med..

[27] Song M.Y., Kang K.H., Lee H., Kim T.I., Koh K. (2022). A Comparative Study of Two Extended Depth of Focus Intraocular Lenses. Eye Contact Lens..

[28] Pedrotti E., Bruni E., Bonacci E., Badalamenti R., Mastropasqua R., Marchini G. (2016). Comparative Analysis of the Clinical Outcomes with a Monofocal and an Extended Range of Vision Intraocular Lens. J. Refract. Surg..

[29] Wu T., Wang Y., Yu J., Ren X., Li Y., Qiu W., Li X. (2023). Comparison of dynamic defocus curve on cataract patients implanting extended depth of focus and monofocal intraocular lens. Eye Vis..

[30] Mencucci R., Favuzza E., Caporossi O., Savastano A., Rizzo S. (2018). Comparative analysis of visual outcomes, reading skills, contrast sensitivity, and patient satisfaction with two models of trifocal diffractive intraocular lenses and an extended range of vision intraocular lens. Graefes Arch. Clin. Exp. Ophthalmol..

[31] Tabuchi H., Tanabe H., Shirakami T., Takase K., Shojo T., Yamauchi T. (2023). Comparison of visual performance between bifocal and extended-depth-of-focus intraocular lenses. PLoS ONE.

[32] Khoramnia R., Baur I.D., Łabuz G., Köppe M.K., Hallak M.K., Auffarth G.U. (2023). Functional outcomes after bilateral refractive lens exchange with a continuous-range-of-vision intraocular lens. J. Cataract. Refract. Surg..

[33] Qiao L., Wan X., Cai X., Vasudevan B., Xiong Y., Tan J., Guan Z., Atchison D.A., Wang N. (2014). Comparison of ocular modulation transfer function determined by a ray-tracing aberrometer and a double-pass system in early cataract patients. Chin. Med. J..

[34] Lee H., Lee K., Ahn J.M., Kim E.K., Sgrignoli B., Kim T.I. (2014). Evaluation of optical quality parameters and ocular aberrations in multifocal intraocular lens implanted eyes. Yonsei Med. J..

[35] Lubiński W., Podborączyńska-Jodko K., Kirkiewicz M., Mularczyk M., Post M. (2020). Comparison of visual outcomes after implantation of AtLisa tri 839 MP and Symfony intraocular lenses. Int. Ophthalmol..

